# Alcohol and cannabis use for pain management: Translational findings of relative risks, benefits, and interactions

**DOI:** 10.1016/j.physbeh.2025.114867

**Published:** 2025-02-27

**Authors:** Sumin Lee, Scott Edwards

**Affiliations:** Department of Physiology and Comprehensive Alcohol-HIV/AIDS Research Center, Louisiana State University Health Sciences Center, 2020 Gravier St. Room 734, New Orleans, LA 70112, USA

**Keywords:** Alcohol, Amygdala, Analgesia, Cannabis, Delta-9-tetrahydrocannabinol, Polysubstance use

## Abstract

Chronic pain affects over 20% of the global population and contributes to the vast burden of psychiatric illness. While effective treatments for chronic pain remain limited, both alcohol and cannabis have been used for centuries to manage pain and closely associated negative affective symptoms. However, persistent misuse of alcohol and/or cannabis in such a negative reinforcement fashion is hypothesized to increase the risk of severity of substance use disorders (SUDs). The current review describes neurobiological evidence for the analgesic efficacy of alcohol and primary cannabis constituents and how use or co-use of these substances may influence SUD risk.

## 1. Introduction

Alcohol and cannabis use are highly prevalent across the world. In the United States (US) alone, 177 million (63%) people aged 12 and older reported alcohol consumption in 2022, which is more than a 10% increase from the previous year [1]. Marijuana/cannabis was the most widely used illicit drug in the US with an estimated 61.9 million (22%) people aged 12 and older reporting use in the past year, a 3% increase compared to the previous year [1].

Alcohol use disorder (AUD) is a medical condition characterized by the inability to control or stop alcohol use despite adverse consequences, and about 11% of the population met the criteria for AUD in 2022 [1]. Severe AUD or alcohol dependence is a chronic, relapsing disease with the emergence of negative emotional states (e.g., depression, anxiety, and pain) during withdrawal and a profound escalation of drinking [2]. As such, motivation for alcohol use is conceptualized to progress from recreational, limited consumption (dominated by positive reinforcement principles) to uncontrolled, escalated drinking in the context of AUD (transition to negative reinforcement) [3].

Negative reinforcement theories of AUD posit that excessive drinking may be driven by the desire to alleviate either specific or widespread stress and withdrawal symptoms. As one example, pain is a negative subjective experience with both somatic and affective components that can have a powerful influence on reward and reinforcement mechanisms that could foster the progression to AUD in vulnerable individuals [4]. While it has long been observed that alcohol administration elevates human pain thresholds [5], withdrawal from chronic alcohol use often results in heightened sensitivity to innocuous (allodynia; Table 1) or noxious (hyperalgesia; Table 1) stimuli as part of a more complex alcohol withdrawal syndrome [6,7]. Evidence suggests that excessive drinking in alcohol-dependent individuals may in part be motivated by the desire to self-medicate and alleviate hyperalgesia and/or pain-related negative affect [8,9]. Acute pain is evolutionarily adaptive as it serves to warn the body about real or potential tissue damage in order to protect the damaged area and prevent further harm. However, chronic pain is a pathophysiological state where pain continues long after the damaged tissue has healed and no longer has a protective function. Chronic pain affects over 50 million individuals in the United States alone [10], a number that will likely increase over the next several decades given the aging population [11]. Chronic pain also contributes to both polysubstance misuse and the development and maintenance of shared or composite substance use disorders (SUDs), including alcohol, cannabis, and opioid use disorders [12,13].

The concurrent (general use) and simultaneous (use at the same time) intake of alcohol and cannabis is of particular concern as they are associated with greater rates of SUDs and increased prevalence of mental illness [1]. With the increasing number of states legalizing the medicinal and recreational use of cannabis, uncovering the neurobiological and biopsychosocial relationships among cannabis use, alcohol drinking, and chronic pain in individuals at risk for SUD has never been more crucial. Thus, the purpose of this review is to provide an overview of the existing literature on the effects of alcohol and cannabis on pain regulation. Specifically, we will review (1) preclinical and clinical laboratory studies examining the analgesic efficacy of alcohol and cannabis, including sex differences, (2) the physiological effects of alcohol and cannabis co-use, and (3) the central brain regions involved in nociceptive signaling that are impacted by alcohol and cannabis use. This review includes studies in adults only and includes both simultaneous use and concurrent use, which will referred to as co-use throughout this review unless the differentiation is relevant to study findings. Articles were identified using PubMed and Google Scholar with search terms cannabis, marijuana, tetrahydrocannabinol (THC), alcohol, co-use, dual use, simultaneous use, concurrent use, pain, anti-nociceptive, and analgesic.

## 2. Analgesic efficacy of alcohol

Alcohol use specifically for pain management is common, with 25% of chronic pain patients reporting alcohol use to treat their pain [14]. Interestingly, problem drinkers report more severe pain symptoms compared to non-drinkers, and 79% of high-risk alcohol users drink alcohol to manage their pain [15,16]. This section will summarize the efficacy and neurobiological mechanisms underlying the analgesic effects of alcohol.

Alcohol was widely used as an anesthetic for its sedative and analgesic effects in the 19th and 20th centuries [17]. Indeed, a meta-analysis of human studies found that alcohol administration results in a significantly higher pain threshold and reduced pain intensity ratings [18]. The increase in empirical pain thresholds after alcohol administration occurred in a dose-dependent manner, where every 0.02% increment in blood alcohol concentration (BAC; equivalent to 1 standard drink) was associated with greater analgesia in relation to pain threshold and pain intensity. Concerningly, effective analgesia is achieved at or above binge drinking levels, which may place individuals at heightened risk for AUD development. Interestingly, alcohol consumption is associated with lower odds of chronic pain episodes as well [19], which may prompt individuals experiencing chronic pain to drink alcohol to self-medicate their pain.

However, the analgesic efficacy of alcohol in individuals varies according to multiple factors. One study found that individuals with a family history of alcoholism and high neuroticism (propensity to experiencing negative emotions) reported significantly more analgesia following exposure to a low alcohol dose [20]. In addition, negative affect is a key supraspinal component of pain processing [21,22], and it has been suggested that people with AUD may be drinking primarily to cope with negative affect [23,24]. Indeed, individuals with depression and anxiety symptoms have significantly greater odds of self-medicating their pain with alcohol and other substances [25]. Family history and depression have long been identified as risk factors for the development of alcohol dependence [26,27]. Another factor that could influence alcohol analgesia is the expectation of such effects. A high expectation of alcohol analgesia positively correlated with increased quantity and frequency of alcohol consumption and chronic pain grade, suggesting that motivation for chronic and heavy alcohol use may become pathologically intertwined with an individual’s desire for pain management over time [28].

Although acute alcohol administration is known to produce a dose-dependent analgesic effect, chronic alcohol consumption may result in hyperalgesia that is particularly evident during withdrawal or abstinence periods [7,8,29]. Previous research demonstrated that patients receiving treatment for AUD exhibit greater pain symptoms with decreased pain tolerance and thresholds during alcohol abstinence [6]. Even in healthy individuals, episodic binge drinking can lead to hyperalgesia symptoms [30]. This may be due to the initial rewarding and analgesic effects of alcohol followed rapidly in time by an opposing dysphoric and hyperalgesic state manifesting during acute alcohol withdrawal [8,31].

Importantly, sex differences in chronic pain experiences are evident [32], and they have long been observed in the context of alcohol use, AUD prevalence, pain, and their intersections [33–35]. Women are more likely to experience chronic pain, more sensitive to pain, and are at a higher risk of developing long-lasting pain after injury [36–38]. A meta-analysis of alcohol consumption and chronic pain has revealed that the association is stronger in females than in males, although this difference is not statistically significant [19]. This trend may be due to women having a higher pain sensitivity, as well as a higher pain threshold and pain tolerance [39]. Multiple mechanisms have been proposed to explain such sex differences in pain, including sex hormone actions, differential endogenous opioid systems, the effects of cognition and affection, and psychosocial factors [32,40]. Another possible explanation includes differential metabolism of alcohol in males versus females. Women tend to display increased alcohol bioavailability after drinking, as they have lower levels of gastric alcohol dehydrogenase activity and first-pass metabolism, as well as slower recovery from alcohol-induced cognitive impairment compared to men [41,42]. These biological, psychosocial, and behavioral factors highlight the complex interplay among alcohol use, pain sensitivity, and chronic pain, as well as the multifaceted nature of sex differences in the relationship between chronic pain and alcohol use.

These findings highlight the importance of considering acute vs. chronic alcohol exposure and individual differences when examining its analgesic efficacy and the potential long-term consequences of alcohol use on both pain sensitivity and pain perception. More studies also need to be conducted to uncover factors that could result in individual differences in the propensity to develop or exacerbate AUD and analgesic efficacy of alcohol in sexual minority and transgender populations. Identifying such factors may facilitate the development of preventative and therapeutic strategies for AUD in such vulnerable populations as well as shed light on key biological and sociological factors related to sex and gender differences in relation to pain outcomes.

Animal studies have provided valuable insights into the neurobiological mechanisms underlying the analgesic effects of alcohol. Preclinical research using rodent models has demonstrated time- and dose-dependent effects of alcohol on pain modulation [43–47], with low to moderate doses often producing analgesic effects through the modulation of neurotransmitter systems such as endogenous opioids and γ-aminobutyric acid (GABA) pathways [48–50]. Importantly, studies have indicated that animals may develop tolerance to the anti-nociceptive effects of alcohol with repeated use [44] or that alcohol efficacy may be diminished over time in the context of chronic pain [46]. As time experiencing pain progresses, this phenomenon may lead to increased alcohol drinking to overcome both physiological and analgesic tolerance.

Alcohol administration increases β-endorphin release in the pituitary gland, the hypothalamus, and other brain regions involved in reward and nociception [51–53] as a consequence of alcohol-induced increases of the hypothalamic corticotropin-releasing factor (CRF). Alcohol-induced release of β-endorphin occurs in a dose-dependent manner where low concentrations induce a higher release of β-endorphin, which is not maintained under prolonged exposure to alcohol [54]. It is this decreased β-endorphin activity after chronic ethanol exposure that may represent one neurobiological mechanism to promote and maintain alcohol consumption through negative reinforcement. After long-term exposure, the central nervous system undergoes adaptive changes to compensate for the effects of chronic alcohol exposure to foster a transition from homeostasis to allostasis [55]. Thus, the abnormalities in distinct neuronal circuits may cause pain and comorbid negative affect to increase craving and motivation to consume alcohol during alcohol abstinence or withdrawal after extended exposure [9].

Since alcohol is a positive allosteric modulator of the GABA_A_ receptor channel, the activation of inhibitory GABAergic signaling in the central nervous system likely plays a central role in mediating the analgesic effects of alcohol [49,56]. When GABA_A_ receptors are acutely exposed to alcohol, a potentiation of GABA-gated current occurs, hyperpolarizing the postsynaptic membrane and decreasing neuronal excitability more than seen with GABA activation alone [57]. Interestingly, flumazenil, a benzodiazepine antagonist, abolishes the anti-nociceptive effects of alcohol in rodents [58], suggesting that GABA_A_ receptors are closely involved in regulating alcohol-induced analgesia.

Somewhat paradoxically, chronic and excessive alcohol exposure results in the progressive development of hyperalgesia (or increased nociceptive sensitivity) in animals [59], representing a profound characteristic of alcohol dependence and withdrawal that may be related to alcohol-induced neuropathy in humans [60]. Indeed, chronic alcohol consumption or exposure induces hyperalgesia in various rodent models [61–63]. This alcohol abstinence-induced hyperalgesia in rodents may be in part mediated by neuroadaptations across a variety of systems. Altered glucocorticoid receptor (GR) activity is thought to arise from dysregulation of the hypothalamic-pituitary-adrenal (HPA) axis, a key stress response system. Chronic alcohol exposure can sensitize central GR signaling, leading to heightened cortisol-related effects during withdrawal, which exacerbates nociceptive processing and amplifies pain sensitivity [64]. Similarly, disruptions in glutamatergic and GABAergic signaling play a critical role, as these systems are essential for maintaining the balance between neuronal excitation and inhibition. Chronic alcohol use increases excitatory glutamate transmission while impairing inhibitory GABAergic signaling [65,66], creating a hyperexcitable state in pain pathways that contributes to hyperalgesia. M-type potassium channels, which normally act to stabilize membrane potential and dampen neuronal excitability, also undergo dysregulation during alcohol abstinence [67]. Their reduced activity can lead to hyperexcitability in pain-processing neurons, further heightening nociceptive sensitivity. Finally, alcohol-induced changes in endogenous neuropeptide systems, such as the dynorphin-kappa opioid receptor (KOR) pathway and the endorphin-mu opioid receptor (MOR) system, may also contribute significantly to hyperalgesia [68]. Fortunately, these recent studies have generated a host of novel medication strategies for targeting pain in the context of AUD.

Previous studies have demonstrated sex differences in alcohol-induced antinociception in animal models that are not always consistent, indicating that alcohol may modulate different types of pain modalities between females and males [50]. Only male rats showed decreased thermal sensitivity in association with increased acute alcohol consumption [69], and our group has also previously found that alcohol produces dose-dependent mechanical antinociception and anti-hyperalgesia in female rats but only anti-hyperalgesia in male rats [46].

With regard to chronic alcohol consumption, only male mice appear to exhibit greater mechanical hyperalgesia while only females exhibit thermal hyperalgesia following withdrawal from chronic ethanol exposure [63]. However, other studies have shown that female rats exhibit greater mechanical hypersensitivity after chronic alcohol exposure compared to males [70–72]. While these discrepancies could be due to species differences, additional studies are needed to uncover the mechanistic basic of sex differences in antinociception and hyperalgesia development in response to both acute and chronic alcohol exposure, as well as consideration of differential alcohol metabolism and alcohol pharmacokinetics in various species and between sexes. However, these initial results indicate how profoundly sex might interact with vulnerability for AUD, especially given how alcohol differentially affects pain perception and negative affect in unique sex-dependent manners.

## 3. Analgesic efficacy of cannabis/THC

Cannabis was originally defined as a set of closely associated terpenoids found only in marijuana plants (including several species of the genus *Cannabis*), particularly delta-9-tetrahydrocannabinol (THC) [73]. Currently, cannabis as a substance refers to any chemical that can activate cannabinoid receptors. Related psychoactive cannabinoid substances have shown potential in the treatment of many chronic pain conditions and have been used for medicinal purposes by licensed practitioners [74]. Endocannabinoids (eCBs) are endogenous lipids that interact with cannabinoid receptors to maintain neuronal homeostasis and confer synaptic plasticity throughout the nervous system [75,76]. The eCB system, comprised of eCBs and their receptors, produces endogenous analgesic effects and modulates nociceptive sensitivity [77, 78]. The two major eCBs anandamide (AEA) and 2-arachidonoyl glycerol (2-AG) are retrograde synaptic messengers that inhibit synaptic transmission [79,80] and cannabinoid receptors CB1R and CB2R are members of the inhibitory G-protein coupled receptor superfamily [81, 82]. CB1 receptors are predominantly found in the central nervous system, while CB2 receptors are commonly found in immune cells (including brain immune cells) and various peripheral organs [83]. The activation of CB1 receptors in the central nervous system inhibits nociceptive signaling pathways, while modulation via CB2 receptors may attenuate neuroinflammatory responses associated with numerous pain conditions [80,84–86].

THC is the main psychoactive component of cannabis and a partial agonist of CB1 receptors [87]. Cannabinoids elicit several physiological responses upon binding to CB1Rs and CB2Rs, such as hypothermia, analgesia, reduced physical activity, and catalepsy [88]. In addition, cannabis also results in increased sensory awareness, nausea relief, and a state of moderate euphoria [89–91], suggesting its potential for dependence and addiction, termed cannabis use disorder (CUD). Cannabidiol (CBD) is another major compound found in cannabis-based plants that may exhibit some therapeutic potential, although its pharmacology appears much more complex. CBD is a non-competitive negative allosteric modulator of CB1Rs in the presence of THC and has a very low binding affinity for either CB1R or CB2R [92]. CBD consumption increases THC plasma concentrations while reducing THC-induced analgesia, indicating antagonistic pharmacodynamic interactions between THC and CBD [93]. A retrospective observational study showed that CBD-rich medications have beneficial effects for pain, anxiety, depression, and epilepsy in the absence of THC, although prolonged use may lead to organ damage [94,95]. For the purpose of this review, the following discussions around the analgesic efficacy of cannabis will focus exclusively on delta-9-THC.

Although over 30% of individuals with chronic pain report using cannabis for pain management [74], human studies investigating the analgesic efficacy of cannabis have yielded mixed results. While some clinical trials and observational studies have reported significant reductions in pain intensity and improved pain-related outcomes with acute cannabis use, others have found minimal to no effects [96–100]. Interestingly, cannabis appears to increase pain thresholds in patients with chronic pain [93] while increasing nociceptive sensitivity under acute pain conditions [100]. Such discrepancies in study findings indicate the complex and differential effects of THC in the context of acute versus chronic pain.

When effective, THC tends to dose-dependently produce analgesia, where higher doses lead to greater pain relief [101,102]. However, participants who experience higher doses of THC (15 mg and 20 mg) are often heavily sedated [101]. THC is often consumed via inhalation of smoke and/or vapor or ingestion of an edible product, and the pharmacokinetics can vary depending on the mode of administration [103, 104]. Its psychoactive effects, including analgesia, reflect plasma THC concentrations [105] and can occur rapidly with 2 to 3 mg of inhaled THC or slowly with 5 to 20 mg of ingested THC in naïve users [104, 106–108]. Importantly, the analgesic effect of cannabis seems to diminish, requiring higher doses over time, suggesting a development of analgesic tolerance with chronic use [109,110]. Doses higher than 20 mg are often associated with its sedative effects [101,111], potentially confounding THC’s analgesic specificity. Thus, the observed results could be due to either an interference of pain perceptions or an indication of the apparent narrow therapeutic window for a non-sedative analgesia for cannabis. These results altogether suggest that cannabis can produce reliable acute analgesic effects among individuals with chronic pain [112]. However, recent studies have revealed that high daily cannabis use is associated with greater pain severity [113,114], highlighting the need for development of cannabis use guidelines and potential limits for the effective treatment of chronic pain.

Tolerance, the loss of therapeutic efficacy with repeated use, is highly undesirable in an analgesic medication and can potentially lead to misuse in the form of escalated intake [115]. Chronic activation of cannabinoid receptors leads to tolerance development, mainly via desensitization and downregulation of CB1Rs [116–118]. In addition, rodent models show dose-dependent signs of physical dependence such as paw tremors when treated with CB1R antagonists [116,118]. Moreover, because THC can produce euphoria and rewarding/reinforcing effects [119,120], the use of cannabis should be carefully considered in treating pain or other medical conditions in individuals with high propensity for drug misuse or a family history of SUDs.

Additionally, variability in individual responses to THC-induced analgesia is multifactorial, influenced by factors such as genetic predisposition [121], previous cannabis exposure [122], and psychological factors [122,123]. Additionally, differences in cannabis strains [96], THC content [124], and route and mode of administration [106,125] may contribute to the variability in pain relief experienced by individuals.

Sex differences are another important factor in describing cannabis use and efficacy [126]. Men consume cannabis in greater amounts and higher rates than women [127,128], possibly contributing to male users showing more profound withdrawal symptoms [129] and higher co-morbid psychiatric disease prevalence [130]. These differences may arise from sex-specific pharmacokinetic factors, including the distribution and metabolism of cannabinoids. Women have a higher percentage of body fat, which serves as a reservoir for lipophilic compounds like THC, leading to prolonged exposure and slower clearance compared to men [126]. Even in cannabis-naïve individuals, men report larger subjective, intoxicating effects of THC compared to women [131]. In contrast, cannabis-naïve women show greater hemodynamic changes and visuospatial memory impairment following cannabis use compared to men [132].

Regarding the sex differences in the analgesic efficacy of cannabis, the findings are mixed. One clinical, double-blind, placebo-controlled, crossover study found that a single dose of nabilone (CB1R and CB2R agonist) produces anti-hyperalgesia to thermal noxious stimuli in women only [133]. A retrospective analysis between the analgesic efficacy of cannabis in daily users from a double-blind, placebo-controlled study showed that cannabis decreases pain sensitivity to cold noxious stimuli in men only [134]. However, because the type of noxious stimuli, dose of cannabis, and the route of administration were different in these two studies, a meaningful, comparative conclusion cannot be made. Altogether, the complex interaction between such factors highlights the need for personalized approaches to cannabis-based pain management.

Animal studies have provided valuable insights into the analgesic properties of THC and its underlying mechanisms in the context of acute, chronic, and neuropathic pain [135–137]. Many studies have shown additive effects of opioids and cannabis in producing analgesia [115], suggesting a close relationship between the endogenous endocannabinoid and opioid systems in modulating pain signaling. Others have found synergistic effects of cannabinoids with gabapentin, acetaminophen, and non-steroidal anti-inflammatory drugs [115], indicating an anti-inflammatory role for cannabinoids to produce analgesia.

Sex differences in cannabis responsiveness have been found in animal models as well. Cannabinoids produce greater analgesia, catalepsy, and locomotor effects [138] along with diminished negative affective-like behaviors in female rats compared to male rats [139]. Another study also showed that female mice experience increased THC-induced antinociception compared to male mice at low and moderate doses [140]. However, female rodents also develop tolerance to the anti-allodynic effects of THC faster than male rats [141]. These sex differences in responsiveness and tolerance may be attributed to variations in pharmacokinetics and central accumulation of THC and its metabolites, where female rats exhibit higher and more prolonged concentrations of the active metabolite 11-hydroxy-THC (11-OH-THC) in the brain following cannabis administration [142].

## 4. Co-use of alcohol and cannabis in the context of pain

Co-use of cannabis and alcohol is widely prevalent in the United States [143]. Cannabis is the second most commonly used drug among individuals with alcohol use disorder (AUD) after tobacco [144], and more than 75% of cannabis users report consuming alcohol [145]. The co-use of alcohol and cannabis is especially prevalent among individuals seeking pain relief of pain-related symptoms [146], and many individuals with chronic pain consume both alcohol and cannabis to manage their symptoms [147,148]. Furthermore, the co-use of alcohol and cannabis is particularly high among young adults [149,150] and individuals with mental health conditions that are often comorbid with chronic pain [151].

Individuals who self-medicate with alcohol and cannabis for alleviation of their chronic pain symptoms may be at an enhanced risk for SUD development [152]. In addition, the analgesic properties of alcohol and cannabis may reinforce their use in individuals with chronic pain, perpetuating a cycle of substance misuse and hyperalgesia [119,120, 153,154]. Studies show that when cannabis and alcohol are consumed during the same occasion, both substances are used at higher quantities and frequencies compared to when used alone [155,156]. Thus, understanding the relationship between alcohol and cannabis, especially in the context of motivated co-use and chronic pain, poses as an important area of needed research.

Limited work exists communicating the analgesic efficacy of alcohol and cannabis co-use. Some studies report enhanced pain relief with co-use, possibly due to increased THC concentration in the blood with alcohol consumption [157,158]. Others may experience adverse side effects such as increased sedation, impaired motor coordination, and cognitive dysfunction [157,159]. The synergistic or antagonistic interactions between alcohol and cannabis in pain regulation remain poorly understood and require further investigation. Additionally, the long-term consequences of co-use, including the possible risk of developing SUDs and exacerbated cross-tolerance or hyperalgesia, must be taken into consideration in clinical practice.

Rates of SUDs and treatment admissions are the highest among co-users of cannabis and alcohol [1]. Individuals with a diagnosis of cannabis use disorder (CUD) are also at a greater risk for the development of AUD [160–163], and cannabis use is associated with increased incidence of AUD [144]. Moreover, a comorbid diagnosis of AUD and CUD is associated with riskier drinking habits and increased cannabis-related problems than having a single diagnosis [164].

**Fig. 1.**
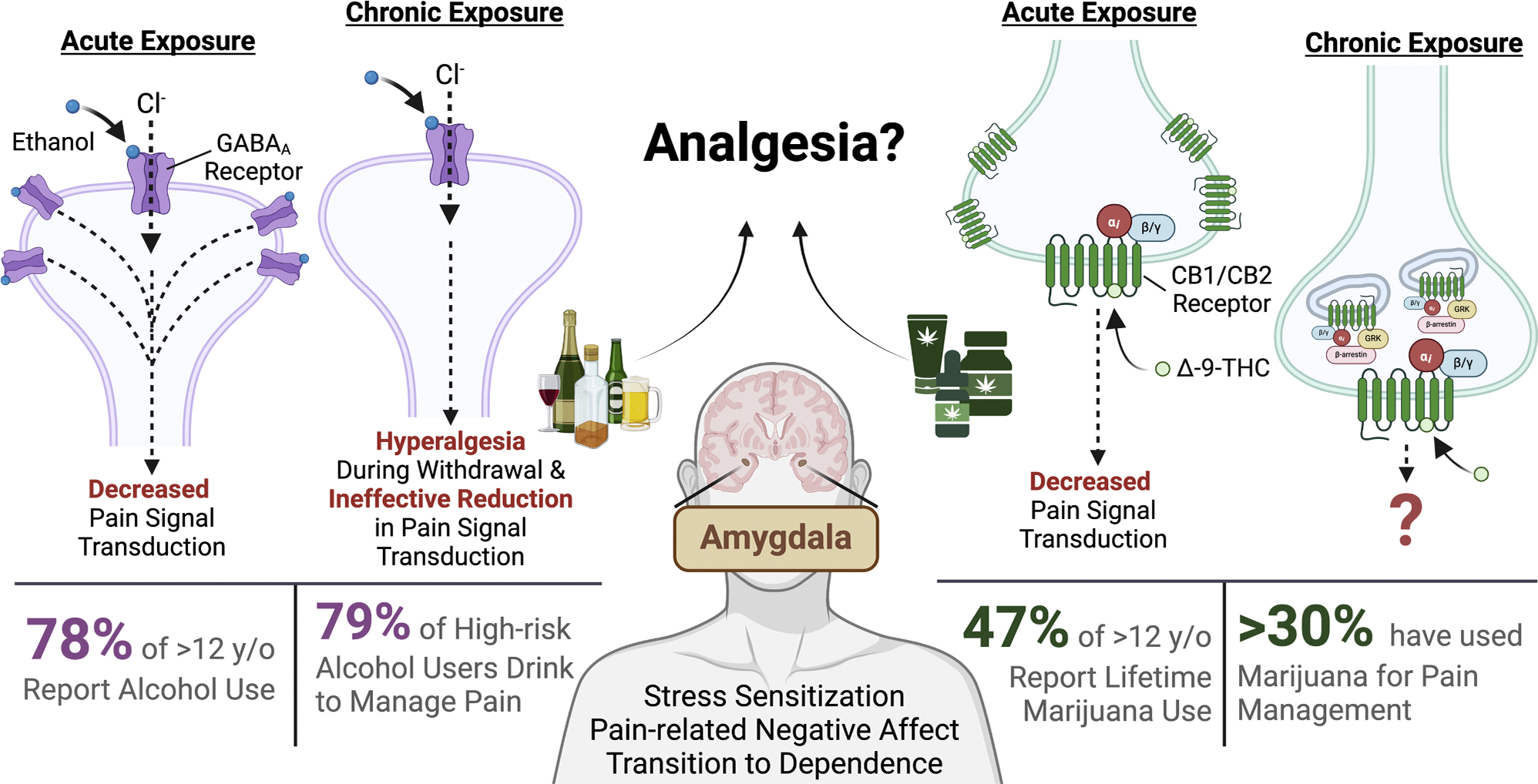
A substantial proportion of individuals with high-risk alcohol use consume alcohol to self-medicate for pain, a pattern also observed among cannabis users. Both substances are known to acutely diminish pain signal transmission; however, chronic alcohol use is associated with heightened pain sensitivity during withdrawal. In contrast, the effects of long-term cannabis use on pain perception remain incompletely understood, as does the combined impact of alcohol and cannabis use on pain symptoms and susceptibility to substance use disorders. This review summarizes the current literature on the roles of alcohol and cannabis in pain regulation. Created in BioRender.

Preclinical research has shown that the eCB system may be involved in alcohol reinforcement and motivation to drink alcohol [165–168]. Some studies show that CB receptor antagonists decrease preference and overall intake of alcohol [165,169,170], while CB receptor agonists and inhibitors of eCB enzymatic degradation increase alcohol-seeking behavior and consumption [166,171]. These findings support potential positive motivational influences between cannabis and alcohol use and serve as important considerations for clinical research in individuals with chronic pain and history of alcohol and cannabis co-use (Fig. 1).

Alcohol dependence is also closely associated with engagement and dysregulation of the eCB system [172]. A state of eCB system deficiency is often observed with chronic alcohol exposure, which is correlated with hyperalgesia to both mechanical and thermal nociceptive stimuli [173]. Indeed, a recent study found that chronic alcohol drinking produced a mechanical hyperalgesia in mice that was alleviated via pharmacological augmentation of 2-arachidonoylglycerol (2-AG) levels, suggesting a specific role for dysregulation of monoacylglycerol lipase activity in alcohol-related pain [174].

## 5. Central brain regions involved in pain processing

**Fig. 2.**
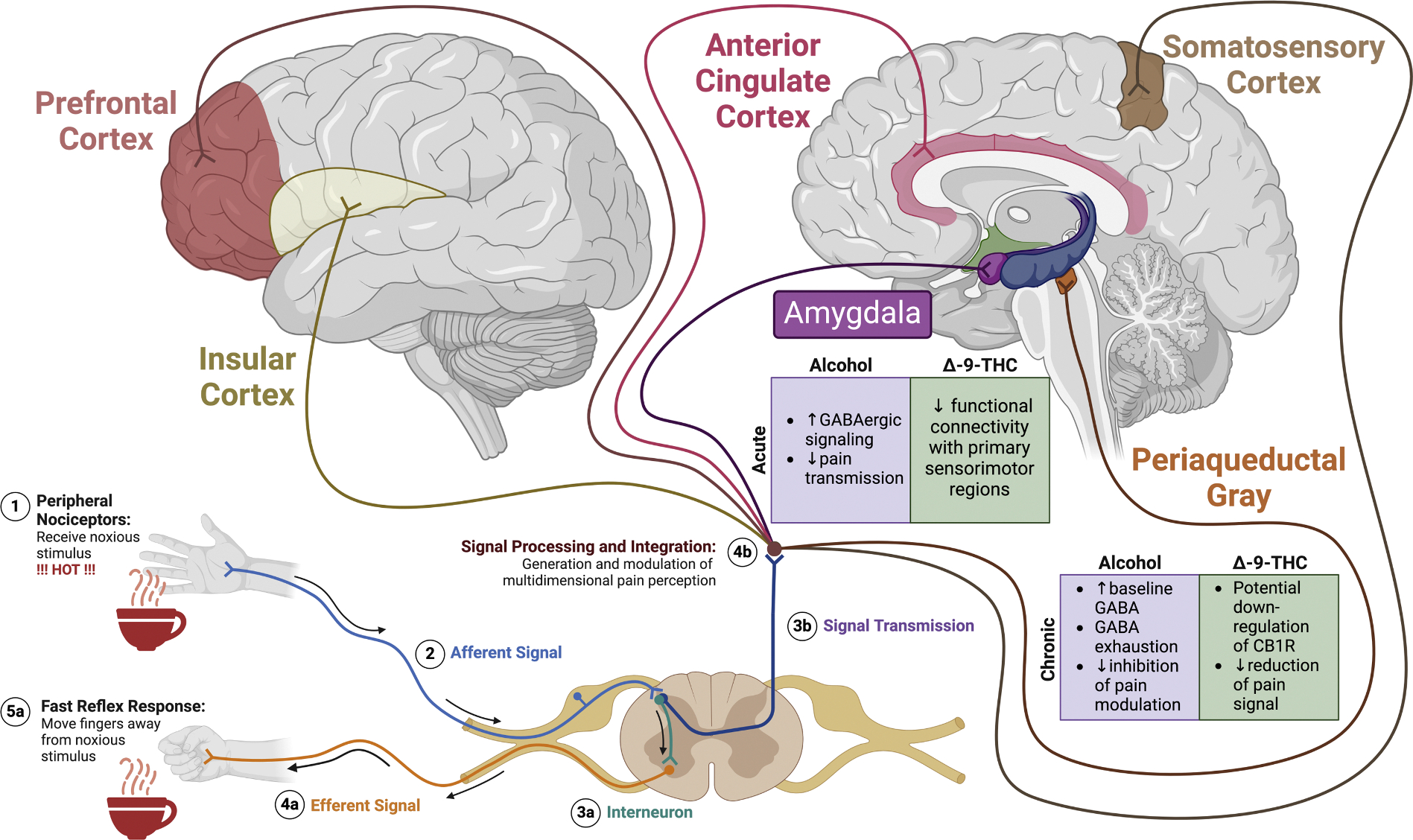
Noxious stimuli are transmitted to elicit both fast reflex responses as well as higher communication across various supraspinal brain regions that are responsible for processing the multidimensional perception of pain, including sensory, cognitive, and motivational aspects. Exposure of nociceptive circuitry to either alcohol or cannabis/THC produces neuroadaptations that likely influence important relationships among pain experiences and motivation for substance use. Created in BioRender.

Pain is defined as “an unpleasant sensory and emotional experience associated with, or resembling that associated with, actual or potential tissue damage” [175]. It is distinct from nociception, as pain includes the somatosensory perception of the noxious stimulus, the processing of negative affect via emotional and motivational circuitry, and an evaluation and modulation of the painful experience via cognitive circuitry [176]. These three components of pain are processed via distinctive areas within the peripheral and central nervous systems (Fig. 2).

Primary afferent nociceptors are activated upon exposure to noxious stimuli, engaging motor and autonomic circuits to produce fast reflex responses [177]. These reflex responses allow for limited exposure to noxious stimuli and injury while the nociceptive information is transmitted to and processed in the forebrain [178,179]. When the information reaches the forebrain, a multidimensional pain perception is generated, facilitating the selection of affective-motivational behaviors. These behaviors are selected based on the features of the sensory-discriminative component and the expected outcomes from the noxious event, such as putting on a band aid to prevent further bleeding. Throughout this process, the cognitive-evaluative component of pain is continuously monitored to alter pain perception based on the experience. If the initially selected behavior fails to alleviate the unpleasantness of pain, another behavior is selected and generated. Oftentimes, conflicting needs may deter individuals from engaging in adaptive behaviors, leading them to endure pain, especially if the pain is perceived as mild [176].

Neuroimaging and neurophysiological studies in humans have shown that noxious stimuli elicit specific connectivity patterns within various central nervous system regions including the somatosensory cortex, thalamus, insular cortex/insula, the prefrontal cortex (PFC), anterior cingulate cortex (ACC), periaqueductal gray (PAG) in the brainstem, and the amygdala [180–182]. Indeed, pain activates brain regions that integrate networks associated with the multidimensional components of pain to regulate multisensory integration, emotion regulation, and cognition and attention among other functions [183, 184].

Of these regions involved in pain processing, the amygdala poses as a pivotal brain region with a significant overlap of pain and addiction processing (Fig. 1), implicating its role in stress sensitization, pain-related negative affect, and the transition to substance use disorders [8,185,186]. The amygdala is a critical part of the limbic system that is located in the medial temporal lobe [187]. It is closely connected with various cortical, subcortical, and brainstem areas that are highly involved in the sensory, cognitive, and affective processing of pain, thereby playing an important role in the modulation of emotional responses to pain and pain-related decision-making [188–191]. The basolateral amygdala (BLA) receives inputs from the thalamus and cortical regions such as the insula, ACC, and the medial PFC (mPFC) for sensory information [192–194] and preferentially responds to noxious stimuli [191]. The output from the BLA to mPFC also provides emotion- and motivation-based information to guide executive functions such as decision-making and behavioral control [182].

The information processed in the BLA is then relayed to the central nucleus of the amygdala (CeA). The CeA mainly serves as the output hub of the amygdala, transmitting information to the forebrain and brainstem regions for pain modulation [195,196]. It mainly receives nociceptive-specific information from the spinal cord and the brainstem and responds mainly to noxious stimuli [197,198]. The CeA can both amplify and attenuate pain-related behaviors and nociception in a cell-type-specific manner, undergoing bidirectional changes in response to an injury [199]. In addition, the CeA plays an important role in alcohol reinforcing effects, representing another possible intersection of central pain modification and alcohol reward [200]. Lastly, the intercalated cells (ITCs), a cluster of inhibitory interneurons located between the BLA and CeA, inhibit CeA neuron excitation of the mPFC neurons important for extinction of negative emotional responses [201–204].

Both alcohol and cannabis use can impact these central brain regions involved in pain processing (Fig. 2). Acute alcohol use facilitates inhibitory GABAergic transmission from the CeA to potentially dampen down pain-related signals [205]. Acute alcohol exposure increases presynaptic GABA release, facilitating inhibitory synaptic transmission in the CeA [206] and BLA [207]. Alcohol-induced hyperalgesia during withdrawal may be due to chronic alcohol exposure resulting in greater baseline GABA release and GABA_A_-mediated inhibition of synaptic transmission from the CeA during withdrawal [208]. This leads to exhaustion of the GABA system when experiencing noxious stimuli, disabling modulation and reduction of pain signaling. A more recent study has shown that chronic alcohol use in the context of alcohol dependence indeed reduces GABAergic signaling from the CeA to the periaqueductal gray (PAG), a brain region that is highly important for descending pain modulation [65]. This indicates that the inhibitory signals from the CeA in descending pain circuitry is reduced, resulting in alcohol-induced hyperalgesia. Central sensitization of nociception may occur in chronic pain states, whereby an increased response is observed along the ascending nociceptive circuitry – including the CeA – leading to broadening of functional nociceptive fields and augmentation of pain processes [188,209,210].

Acute THC consumption increases activity in the amygdala in response to noxious stimuli, and this effect was positively correlated with the analgesic properties of THC [211]. Interestingly, THC can elicit pain dissociative effects, where the unpleasantness – or negative affective salience – is reduced upon consumption of THC without affecting the intensity of pain. THC decreases functional connectivity between the amygdala and primary sensorimotor regions during acute pain states, contributing to its pain dissociative effects [211]. Limited literature exists in terms of how chronic consumption of THC alters neuroadaptations within the amygdala to alter pain perception. Chronic administration of THC and other CB1R agonists have been shown to induce downregulation of CB1R in the amygdala [212–214]. However, the effects of CB1R downregulation within the amygdala on nociceptive signaling or pain experiences are unknown.

## 6. Conclusion

In summary, this review has highlighted the complex interactions among alcohol, cannabis/THC, and pain regulation. Both alcohol and cannabis are widely used analgesics, and understanding their effects on pain regulation is essential given their significant and potentially central roles in pain management as well as the development or exacerbation of SUDs.

Alcohol has demonstrated analgesic effects in both human and animal studies, with acute administration leading to temporary pain relief. However, chronic alcohol consumption may decrease pain thresholds, contributing to the development of chronic pain conditions. The analgesic efficacy of alcohol may vary among individuals, influenced by family history of alcohol misuse, expectation of alcohol analgesia, and sex [20,28,46]. Animal studies have highlighted several neurobiological mechanisms underlying alcohol-induced analgesia such as modulation of the endogenous opioid system and GABAergic signaling in various cortical and subcortical brain regions [58,68,180–182].

Similarly, cannabis, particularly delta-9-THC, has garnered attention for its analgesic properties. Although studies have shown mixed results regarding the analgesic efficacy of cannabis and the effects of chronic cannabis use are not well understood [96–100], THC has significant potential in becoming an effective and useful therapeutic strategy in managing chronic, intractable pain [215].

The co-use of alcohol and cannabis presents additional challenges, with potential implications for pain management and SUDs [216]. Co-use of these substances is highly prevalent among individuals seeking pain relief [146], yet the efficacy and safety of their combined use remain poorly understood. Likewise, sex differences in the analgesic effects of alcohol and cannabis further complicate our understanding of the underlying neurobiological mechanisms.

Lastly, chronic alcohol and cannabis use can impact several central brain regions involved in pain processing, contributing to altered pain sensitivity [6,77,78]. Dysregulation of neurobiological substrates in regions such as the amygdala, insula, and the anterior cingulate cortex may explain the observed changes in pain perception associated with excessive alcohol and cannabis use [217].

Overall, this review highlights the importance of further research to understand the complex relationships among alcohol, cannabis/THC use, and pain. With growing public health concerns associated with the intersection of substance use disorders and chronic pain, understanding these mechanisms is crucial for the development of safer and more effective pain management strategies.

## Figures and Tables

**Table 1:** Definition of terms frequently mentioned throughout the review.

Term	Definition
**Pain**	Multidimensional experience (including sensory and emotional) of internal or external noxious stimuli
**Nociception**	Sensory experience of noxious stimuli
**Analgesia**	Subjective pain relief
**Allodynia**	Increased sensitivity to non-painful or innocuous stimuli
**Hyperalgesia**	Increased sensitivity to painful or noxious stimuli
**Anti-allodynia**	Condition or treatment that reduces or counteracts the state of “allodynia”
**Anti-hyperalgesia**	Condition or treatment that reduces or counteracts the state of “hyperalgesia”
**Anti-nociception**	Condition or treatment that reduces or counteracts the sensory transmission and/or perception of pain signals

## Data Availability

No data was used for the research described in the article.
